# Case Report: Intraoperative Fascial Traction for Increasing Intra-Abdominal Volume in Loss-of-Domain Incisional Hernias: A Report of Two Cases

**DOI:** 10.3389/jaws.2025.14283

**Published:** 2025-03-14

**Authors:** Hakan Gök

**Affiliations:** Hernia Istanbul, Comprehensive Hernia Center, Istanbul, Türkiye

**Keywords:** incisional hernia, loss of domain, intraoperative fascial traction, abdominal compartment syndrome, complex hernia

## Abstract

**Introduction:**

The primary goal in incisional hernia repair is achieving primary fascial closure and reinforcing the area with a synthetic mesh. However, when Loss of Domain (LoD) is present, serious complications such as intra-abdominal hypertension (IAH) and abdominal compartment syndrome (ACS) may arise. Various strategies have been employed to overcome these challenges and increase the reduced intra-abdominal volume, including preoperative botulinum toxin (BTA) injection, progressive pneumoperitoneum (PPP), various component separation techniques, and their combinations. Intraoperative fascial traction (IFT) has recently been added to this armamentarium. The two cases presented here aim to demonstrate the potential benefits of this innovative technique and offer a different perspective to surgeons dealing with such challenging cases.

**Presentation of Cases:**

The two patients presented here had previously undergone open umbilical hernia repair with mesh—one 17 years ago and the other 5 years ago—both of whom experienced recurrence and developed LoD over time. In both cases, IFT was successfully performed, resulting in an uneventful recovery.

**Discussion:**

The repair of incisional hernias accompanied by LoD presents significant challenges. In managing these cases, it is essential not only to optimise the patient preoperatively but also to employ interventions aimed at increasing intra-abdominal volume. In recent years, the intraoperative fascial traction (IFT) technique has emerged as a valuable tool in complex incisional hernia repairs. This technique not only facilitates primary fascial closure but also significantly increases intra-abdominal volume, potentially reducing the risks associated with intra-abdominal hypertension and compartment syndrome.

**Conclusion:**

IFT offers promising advantages in the repair of incisional hernias with LoD, as it addresses the dual challenge of achieving primary fascial closure and restoring intra-abdominal volume. The two cases presented highlight the potential of this innovative technique in achieving successful outcomes. However, further research and larger studies are needed to fully establish its efficacy and long-term benefits in this challenging patient population.

## Introduction

The treatment of incisional hernias with an associated loss of domain (LoD) presents significant challenges. These patients often suffer from comorbidities such as diabetes, obesity, chronic obstructive pulmonary disease (COPD), and cardiac conditions, which individually or in combination can complicate hernia repair [1–5]. Preoperative optimisation, including weight reduction, diabetes control, smoking cessation, and respiratory physiotherapy, is crucial in improving outcomes [5–7].

The primary goal in hernia repair is to achieve a durable and recurrence-free outcome. In incisional hernias, this goal is best accomplished by ensuring the closure of the anterior fascia, followed by reinforcement with a mesh, which minimises complications and recurrence [8–10]. However, in cases where LoD is present, the situation becomes more complex [11].

LoD refers to a condition where a significant portion of intra-abdominal organs resides outside the abdominal cavity within the hernia sac, leading to a reduced intra-abdominal volume. Preoperative imaging with a CT scan and volumetric analysis are essential to assess the presence and extent of LoD [12, 13]. In these patients, the challenge lies in reintegrating the displaced organs into the original abdominal cavity without causing intra-abdominal hypertension (IAH), which can impair organ perfusion and pulmonary function or, in severe cases, lead to abdominal compartment syndrome (ACS) [14–18].

To address these challenges, various techniques have been developed to increase intra-abdominal volume. Preoperative interventions such as botulinum toxin A (BTA) injection, progressive pneumoperitoneum (PPP), and anterior or posterior component separation can be employed individually or in combination based on patient-specific needs [19–28].

In recent years, intraoperative fascial traction (IFT) has emerged as a promising technique for managing patients with large defects and LoD. This approach not only facilitates primary fascial closure but also contributes to increasing intra-abdominal volume, thereby mitigating the risks of postoperative complications [29–31].

In this report, we present two case studies of patients with incisional hernias and LoD, where IFT was successfully employed to enhance intra-abdominal volume and achieve primary fascial closure.

## Case Reports

### Case 1

**FIGURE 1:**
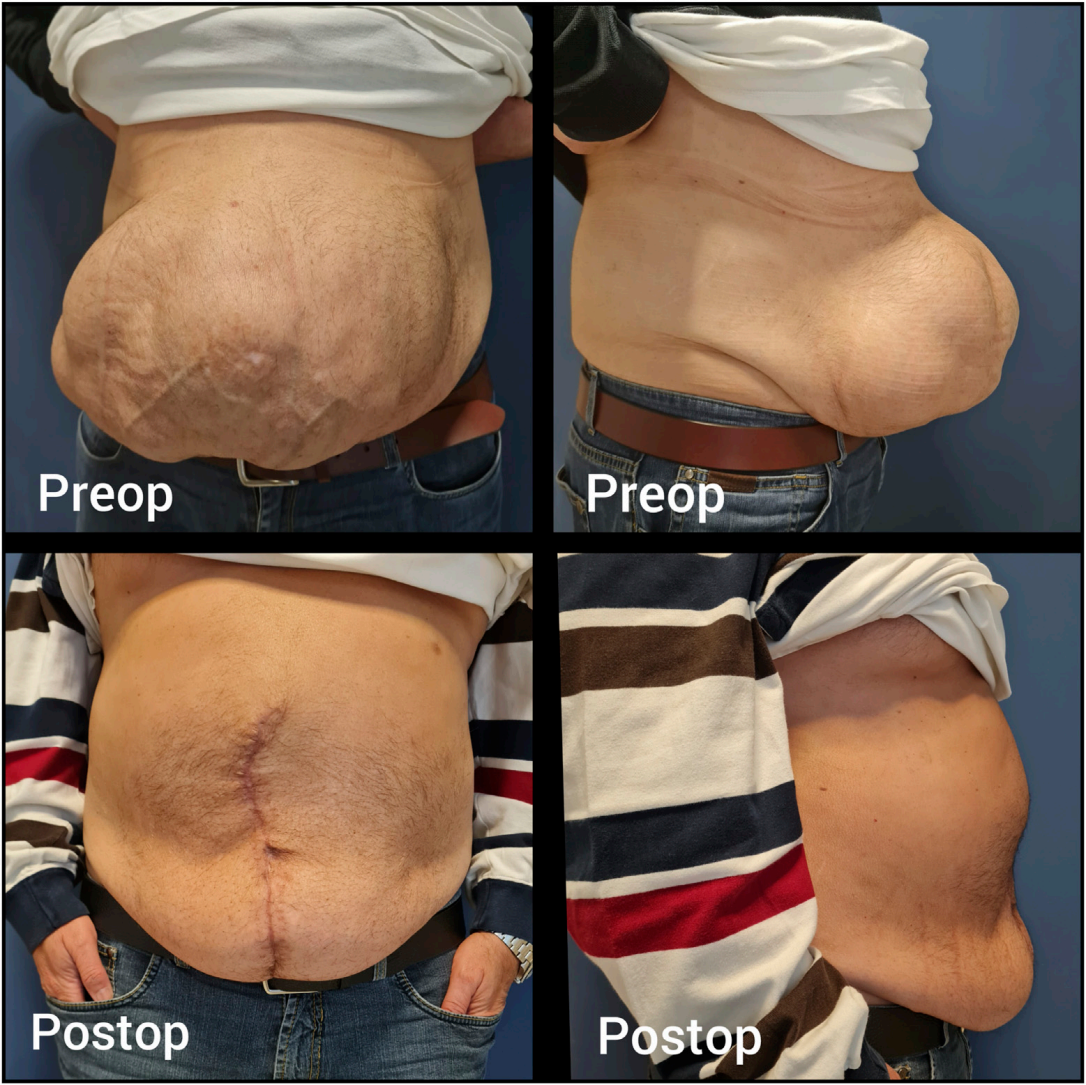
Case 1 - Preoperative and postoperative clinical appearance.

**FIGURE 2:**
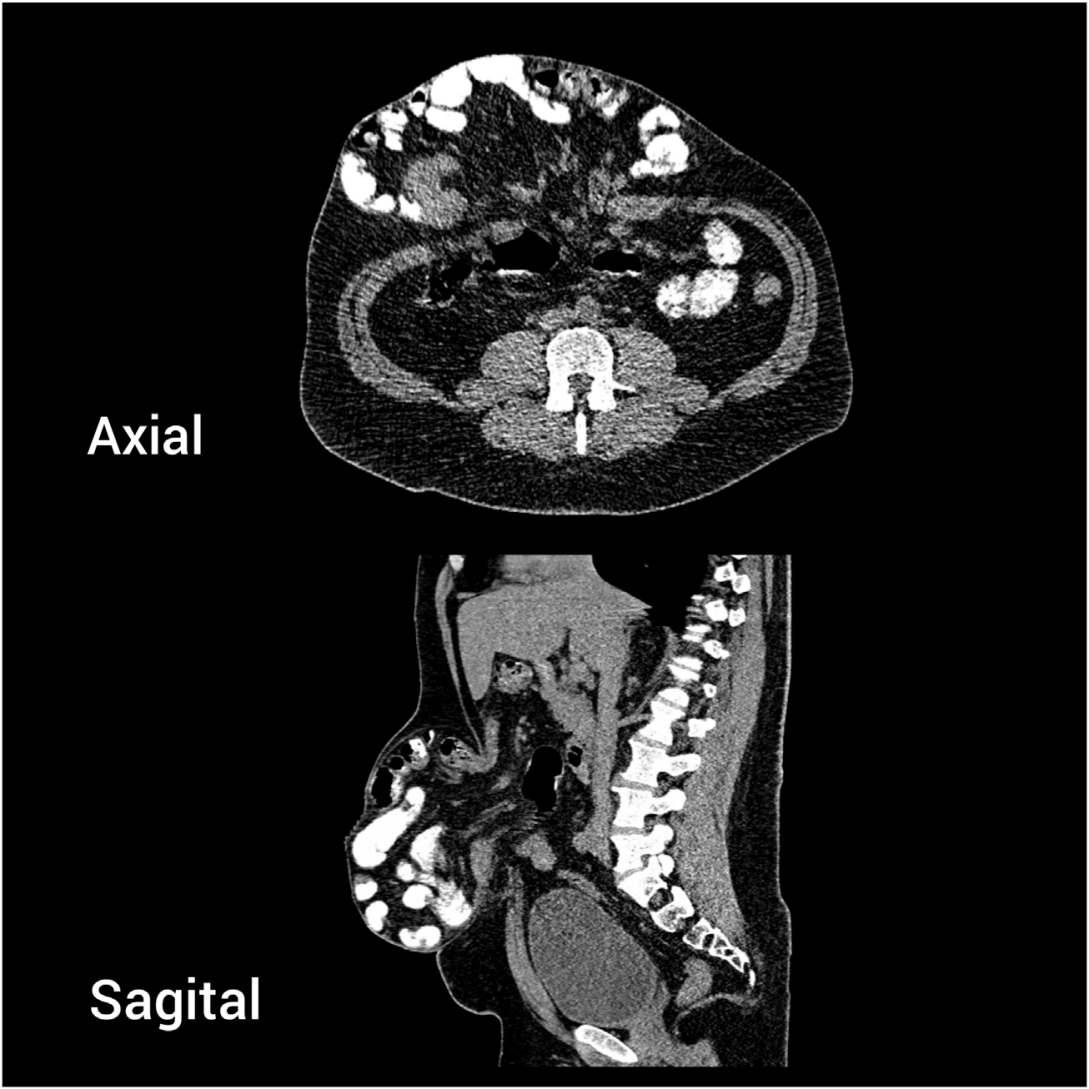
Case 1 - Preoperative CT scan. Axial and sagittal view.

A 49-year-old male patient underwent open umbilical hernia repair with mesh 17 years ago. The hernia recurred shortly after surgery and progressively enlarged over time. The patient had a BMI of 30, type 2 diabetes, and a history of smoking one pack of cigarettes per day. Physical examination revealed a large, irreducible recurrent umbilical hernia (Figure 1). CT imaging showed a 6 × 7 cm hernia defect and a large sac containing small bowel loops under the skin, resembling an hourglass appearance (Figure 2). The Tanaka Index was calculated at 30%.

One month prior to surgery, 200 U of botulinum toxin A (Botox^®^, Allergan Inc., United States) was injected bilaterally under ultrasound guidance into the external oblique (EO), internal oblique (IO), and transversus abdominis (TA) muscles. The patient was advised to quit smoking. However, a urinary nicotine test was not performed before surgery.

During surgery, complete adhesiolysis was performed, followed by a Rives-Stoppa dissection. A 15 × 20 cm medium-weight macroporous mesh was placed in the retrorectus space. IFT using the Fasciotens device (Fasciotens^®^ GmbH, Germany) was applied, enabling successful primary fascial closure without pathological increases in intra-abdominal pressure (IAP). This was checked on the anaesthesia machine by monitoring the peak plateau pressures. The patient developed postoperative paralytic ileus, which resolved with non-operative operative treatment. The patient was discharged without any other complications on postoperative day 5. At the 1-year follow-up, no recurrence was observed (Figure 1).

### Case 2

**FIGURE 3:**
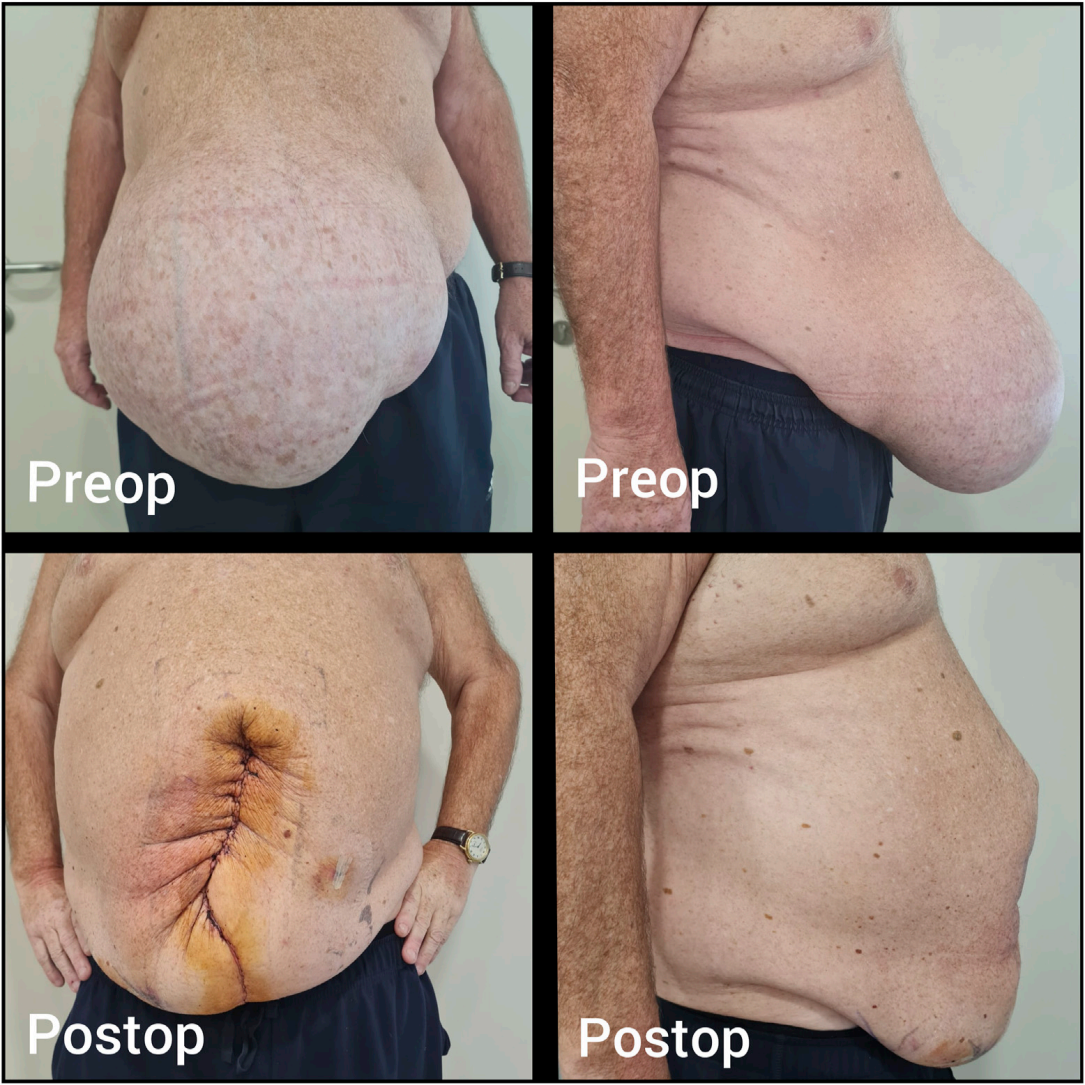
Case 2 - Preoperative and postoperative clinical appearance.

**FIGURE 4:**
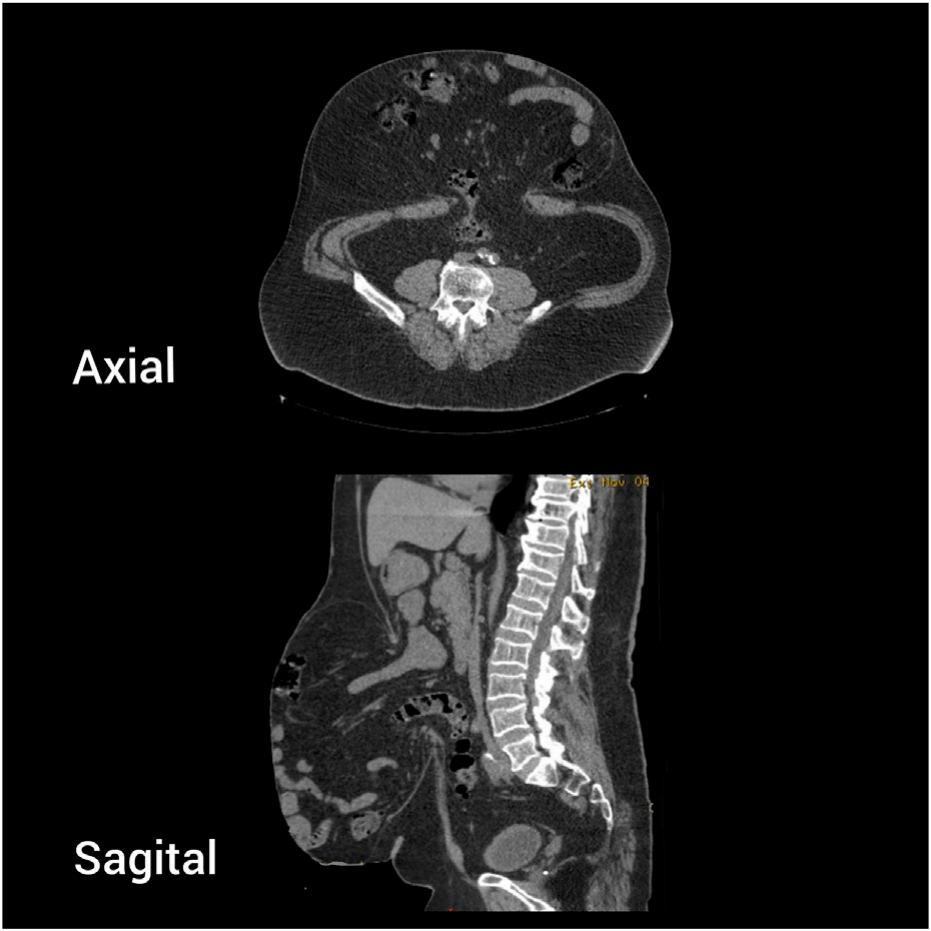
Case 2 - Preoperative CT scan. Axial and sagittal view.

A 64-year-old male patient underwent open umbilical hernia repair with mesh 5 years ago. Recurrence occurred 2 months after surgery, and the hernia progressively enlarged over time. The patient had a BMI of 36.7 and well-controlled hypertension on medication. Physical examination revealed a large, irreducible recurrent umbilical hernia (Figure 3). CT imaging showed a 9 × 12 cm hernia defect and a large sac containing small bowel loops under the skin (Figure 4). The Tanaka Index was calculated at 51%.

One month prior to surgery, 200 U of botulinum toxin A (Botox^®^, Allergan Inc., United States) was injected bilaterally under ultrasound guidance into the EO and IO muscles.

During surgery, complete adhesiolysis was performed, followed by a Rives-Stoppa dissection. An 18 × 30 cm medium-weight macroporous mesh was placed in the retrorectus space. IFT using the Fasciotens device (Fasciotens^®^ GmbH, Germany) was applied, allowing successful primary fascial closure without pathological increases in IAP. The patient was discharged without complications on postoperative day 1. At the time of writing, the patient was in the first postoperative week, with no complications or recurrence observed (Figure 3).

## Discussion

Incisional hernias represent one of the most challenging aspects of abdominal wall hernias. They are not static; they grow over time and significantly impair quality of life [1, 2]. Their repair is complex, with recurrence rates reported as high as 5%–63% [1, 3, 10].

The primary method for reducing recurrence and complications in incisional hernia repair is achieving primary fascial closure [6, 8–10]. Additionally, reinforcement of the repair with a mesh placed in the retromuscular plane significantly reduces recurrence rates. However, in large hernias with LoD returning herniated organs to the abdominal cavity may lead to IAH, potentially resulting in ACS [14–18]. This pathological condition can impair respiratory function, compromise organ perfusion, and may lead to fatal outcomes.

To prevent this, various options are available to increase intra-abdominal volume. These include the use of BTA, which relaxes and elongates the external oblique (EO), internal oblique (IO), and transversus abdominis (TA) muscles, thus increasing abdominal volume [19–23] Although BTA provides a limited volume increase, it significantly contributes to achieving primary fascial closure by simplifying the complexity of the repair, it may downstage the procedure, reducing the need for anterior or posterior component separation (PCS) and transversus abdominis release (TAR), and allowing the repair to be completed with the Rives-Stoppa technique alone [9, 21]. BTA application has become nearly standard for midline hernias classified as EHS W3 (wider than 10 cm) [32]. In our two cases, we utilised BTA both to assist with primary fascial closure and increase intra-abdominal volume.

Another effective method for increasing intra-abdominal volume is the use of component separation techniques (CST). Two primary techniques are widely practiced today: Ramirez’s anterior component separation (ACS) [27] and Novitsky’s PCS with TAR [8]. Both techniques are effective in achieving primary fascial closure in midline hernias. However, they are technically demanding. While ACS carries risks of surgical site occurrences (SSO) such as hematoma, seroma, infection, and skin necrosis, PCS-TAR may result in seroma, infection, or challenging-to-repair EIT Ambivium hernias [10, 33–38]. Therefore, expertise in these techniques and careful case selection are essential.

Progressive pneumoperitoneum (PPP) is another effective method to increase intra-abdominal volume [24–26, 39]. In this technique, a catheter is placed into the abdominal cavity, and ambient air is gradually insufflated over 7–10 days preoperatively to expand the abdominal cavity. While effective, PPP is associated with significant complications such as patient intolerance, respiratory insufficiency, bowel injury, and thromboembolic events [26, 39, 40]. It is also time-consuming and costly.

Recently, the use of IFT has gained popularity, with multicenter studies reporting successful outcomes, particularly in achieving primary fascial closure [29–31]. This technique involves placing 12 traction sutures on the medial edge of each rectus muscle and applying a vertical force (12–20 kg) over 30 min, progressively medializing the rectus complex while also increasing intra-abdominal volume.

In our two cases with midline incisional hernias and LoD, we successfully utilised IFT. For the first case, where the defect was only 6 cm wide, one might argue that it could have been easily closed without requiring any additional intervention to achieve the goal. However, the focus was solely on increasing volume. In the second case, both volume increase and primary fascial closure were achieved without causing IAH.

During retrorectus dissection, when the posterior rectus sheath is separated from the muscle, the rectus muscles lose their cylindrical structure, elongate, and expand the retrorectus space. Therefore, as observed in the second case, the available space for mesh placement is greater than the preoperatively measured rectus widths on the CT scan. However, for a 9 cm defect, an 18 cm wide mesh may appear to provide insufficient overlap. In cases of large defects, there is an ongoing debate among hernia surgeons regarding the necessity of posterior component separation and TAR to achieve adequate mesh overlap. While some advocate for TAR, other centers have reported excellent outcomes without it, and both approaches have been supported by published data [8, 31, 39].

IFT is an easy-to-perform and effective technique. No significant complications specific to the technique or device have been reported to date [41, 42]. It can be combined with BTA, CST, and PPP techniques.

It is essential to recognise that no single technique can be universally applied to all hernias. Tailoring is key. Each case must be evaluated individually to determine the most suitable approach, utilizing one or a combination of the aforementioned techniques to optimise patient outcomes.

### Limitations

This study presents the early outcomes of two cases, with one patient having a follow-up period of 1 year and the other only 1 week. Given the limited sample size and short follow-up duration, these findings cannot be generalised. Further studies with larger cohorts and long-term follow-up are needed to draw more definitive conclusions.

## Conclusion

The management of incisional hernias, particularly those with large defects and LoD, requires a comprehensive and individualised approach. Achieving primary fascial closure remains the cornerstone of repair, but addressing the dual challenges of fascial closure and restoring intra-abdominal volume necessitates the integration of innovative techniques such as BTA, CST, PPP, and IFT.

IFT, in particular, offers promising advantages by effectively increasing intra-abdominal volume and facilitating primary fascial closure with minimal complications. The two cases presented underscore the potential of this innovative technique in achieving successful outcomes. By tailoring the surgical approach to the specific needs of each patient, combining one or more of these advanced techniques, surgeons can optimise outcomes, reduce recurrence rates, and improve overall patient quality of life.

However, further research and larger studies are essential to fully establish the long-term efficacy and benefits of IFT in this challenging patient population.

## Data Availability

The raw data supporting the conclusions of this article will be made available by the authors, without undue reservation.

## Abbreviations

LoD, Loss of Domain; IFT, Intraoperative Fascial Traction; BTA, Botulinum Toxin A; CT, Computerised Tomography; PPP, Progressive Penumo Peritoneum; CST, Component Seperation Technique; ACS, Anterior Component Seperation; PCS, Posterior Component Seperation; TAR, Transeversus Abdominis Release; IAH, Intraabdominal Hypertension; COPD, Chronic Obstructive Pulmonary Disease; ACS, Abdominal Compartment Syndrome.
